# Editorial: Recent Advances in the Genetics of Osteoporosis

**DOI:** 10.3389/fendo.2021.656298

**Published:** 2021-03-12

**Authors:** Jonathan H. Tobias, David Karasik

**Affiliations:** ^1^Musculoskeletal Research Unit, Translational Health Sciences, Bristol Medical School, University of Bristol, Bristol, United Kingdom; ^2^MRC Integrative Epidemiology Unit, Bristol Medical School, University of Bristol, Bristol, United Kingdom; ^3^Azrieli Faculty of Medicine, Bar-Ilan University, Safed, Israel

**Keywords:** genome wide association studies (GWAS), mouse model, zebrafish, multi-omics, BMD

## Introduction

The last few years have seen considerable advances in our understanding of the genetic factors influencing osteoporosis, driven by a range of break-throughs. The seven papers comprising this Research Topic together provide a timely update, describing new insights into the genetic architecture of osteoporosis, application of genetic findings to study causal inference, and state-of-the-art approaches to functional genomics, paving the road for multi-omic applications.

## Genetic Architecture of Bone Mass and Bone Fragility

The genetic architecture of osteoporosis comprises mutations affecting single genes responsible for rare monogenic causes of osteoporosis, and common genetic variants representing genetic susceptibility factors for osteoporosis in the wider population. Makitie et al. discuss how recent advances in genetic methodology have led to a rapid increase in identification of monogenic causes of osteoporosis and related conditions associated with low bone mass and/or increased bone fragility. Osteogenesis Imperfecta (OI), the most common monogenic cause of bone fragility, is due to a defect in bone extracellular matrix, with 85% of cases harboring a mutation in type I collagen. Many other genes are now recognized to cause an OI-like skeletal disorder. Some of these perturbate type I collagen function, such as cartilage-associated protein (*CRTAP*), while others may act by impairing bone mineralization, as proposed for Plastin 3 (*PLS3*). There has also been considerable interest in the discovery of mutations which impair osteoblast differentiation and function, not the least since these may also prove useful therapeutic targets for osteoporosis in the wider population. These include genes involved in WNT signaling, which has an important role in skeletal homeostasis, such as *WNT1*, and the WNT inhibitor frizzled-related protein 4 (*SFRP4*); mutations in both these genes being implicated in rare monogenic cases of osteoporosis.

The paper by Gregson and Duncan provides a comprehensive review of disorders associated with high bone mass (HBM). Even having excluded secondary causes such as degenerative changes, unexplained HBM is not uncommon (prevalence approximately 0.2%). Several very rare mutations underlying HBM have been described, which cause a generalized increase in bone mass as a result of increased bone formation, and a reduction in bone fragility and fracture risk. These mainly arise from mutations leading to increased activation of the WNT/β-catenin signaling pathway. Sclerosteosis, van Buchem’s disease, *LRP4* HBM, *LRP5* HBM, and *LRP6* HBM are all thought to involve this mechanism. Other signaling pathways may also contribute to HBM, as exemplified by HBM arising from a mutation in *SMAD9*, part of the TGF-β superfamily. Several monogenic HBM conditions are also recognized where increased bone mass arises from defective bone resorption, and is associated with increased bone fragility, such as osteopetrosis. In the great majority of HBM cases, no underlying monogenic disorder is evident. Although cases of unexplained HBM were found to be enriched for common high BMD alleles identified in GWASs, this does not exclude a role of rarer mutations yet to be discovered. Given the successful translation from identification of the genetic basis of sclerosteosis to new therapy in the case of Romosozumab (1, 2), further genetic dissection of HBM cases may provide additional benefits for human health.

In terms of polygenic disease risk in osteoporosis, the paper by Koromani et al. describes recent advances in our understanding of the genetic architecture of fracture risk, focusing on common variants as identified from genome wide association studies (GWAS). The article reviews genetic factors found to be associated with fracture risk, after comparing individuals with and without a history of fracture, as well as endophenotypes related to fracture risk including bone mineral density (BMD) measured by DXA scans, and BMD estimated from heel ultrasound (eBMD). To date, the majority of susceptibility loci for fracture have also been implicated in BMD. The authors anticipate that further progress will be helped by improved phenotyping. For example, whereas the great majority of GWAS fracture studies combine all fracture types, fractures at specific sites, like the spine, may have a specific genetic architecture. Progress may also be achieved in extending GWAS to endophenotypes other than BMD. A number of advanced imaging methods have been developed which are capable of ascertaining detailed components of bone structure, which may also have a specific genetic architecture, though a challenge is to assemble large enough study samples to conduct well powered GWASs.

## Studies of Causal Inference

The paper by Zheng et al. discusses the application of common susceptibility loci to examine causal inference using Mendelian Randomization (MR). It provides a number of examples where MR has been applied to examine risk factors for osteoporosis, using either BMD or fracture risk as the outcome. This paper also reviews the main limitations and assumptions involved in MR, such as horizontal pleiotropy, whereby the genetic instrument is related to the outcome *via* a separate pathway to the exposure. A number of methods are discussed to exclude pleiotropy, including the use of bi-directional analyses. If bi-directional causal effects are observed, these usually reflect pleiotropy, for example as a result of genetic correlation between the exposure and outcome due to shared genetic instruments resulting from common biological pathways. That said, instances of true bi-directional pathways may exist, such as a proposed positive effect of BMD on sclerostin, whereas sclerostin also exerts a negative effect on BMD, possibly as part of a negative feedback loop. As well as examining causal relationships for associations initially identified in conventional epidemiological studies, more recent applications of MR are also discussed, such as hypothesis-free approaches to examine causal effects of a given risk factor on a range of outcomes, and application of MR for target validation and drug discovery.

## Functional Genomics Studies

Three articles in this Research Topic discuss recent advances in functional studies aiming to provide a basis for translation of human genetic studies into new treatments for osteoporosis. Maynard and Ackert-Bicknell discuss the availability of data from mouse models, as well as other online resources such as tissue expression panels and expression quantitative trait loci (eQTLs). The authors point out that over 500 susceptibility loci have been identified for osteoporosis, however these causative variants nearly all act to alter gene expression rather than representing the actual causative gene. Identifying the genes responsible for mediating the effects of genetic susceptibility loci on BMD is a prerequisite for identifying potential drug targets for new osteoporosis treatments, remains a major goal of functional genomic studies. Mouse models have proven enormously helpful in this regard, by providing a means of characterizing the function of candidate genes through studying the skeletal phenotype of mice where these have been deleted. The repertoire of mice with specific gene knockouts has increased massively over recent years, due to the work of the International Mouse Phenotyping Consortium (IMPC), which ultimately aims to making embryonic stem cells carrying a knockout allele for all protein coding genes. Mice generated from this program undergo phenotypic screens including limited assessment of skeletal phenotype, with more detailed skeletal phenotyping undertaken by BoneBase, and the Origins of Bone and Cartilage Disease (OBCD) projects, based in the US and UK respectively.

Bergen et al. review the emerging use of zebrafish as an animal model in functional follow up studies of osteoporosis. Zebrafish are vertebrates which show strong similarities in their skeletal physiology to mammals, and are highly suited to genetic studies since constructs which modify the genome can be directly injected into embryos at the single cell stage. Bone formed in zebrafish has the same skeletal cell types and modes of regulation as higher vertebrates, making them suitable for studying processes involved in osteoporosis, which can be carried out dynamically and imaged *in vivo*, for which a range of fluorescent reporter constructs are available. Several imaging methods have been applied to zebrafish which together enable highly detailed assessment of skeletal morphology. A number of zebrafish with mutations in skeletally relevant genes have been studied which re-capitulate a range of skeletal disorders, including osteoporosis and OI. As well as helping to identify causative genes by evaluating the effect of their deletion on the skeleton using methods such as CRISPR/Cas9 editing, zebrafish can be applied to osteoporosis genetics research by providing high throughput assays for compounds which target these genes, based on harvesting and culture of osteoblasts from elasmoid scales.

The final article covers proceedings from a workshop held by the Royal Osteoporosis Society in the UK to review opportunities and challenges in functional genomics research in osteoporosis (Tobias et al.). One of the main conclusions from the workshop is that whereas many promising genetic signals have been identified for osteoporosis, to date it has only been possible to interrogate a small fraction of them in functional studies. Whereas financial and manpower resources remain an important limiting factor, functional studies in osteoporosis genetics will benefit from an expanding repertoire of on-line resources, such as the IFRMS knowledge portal which aims to bring together all relevant functional data (https://msk.hugeamp.org/). In addition, a number of multi-omic resources are available which, with the application of causal inference methods described above, can be applied to identify causative genes responsible for genetic association signals. The paper concludes that a roadmap of functional assessments needs to be established, aiming to integrate multi-omic resources with datasets from human genetics and animal models, in order to translate the wealth of genetic discoveries into new therapies for osteoporosis.

## Author Contributions

All authors contributed to the article and approved the submitted version.

## Conflict of Interest

The authors declare that the research was conducted in the absence of any commercial or financial relationships that could be construed as a potential conflict of interest.
